# Testing the long-run neutrality and superneutrality of money in a developing country: Evidence from Iran

**DOI:** 10.1016/j.mex.2021.101251

**Published:** 2021-01-29

**Authors:** Nasim Iranmanesh, Sayyed Abdolmajid Jalaee

**Affiliations:** a Department of Economics, Faculty of Management and Economics, Shahid Bahonar University of Kerman, Kerman, Iran; b Shahid Bahonar University of Kerman, Afzalipour Square, 22 Bahman Boulevard, Kerman 7616914111, Iran

**Keywords:** Macroeconomics, Monetary policy, Money neutrality, Lee-Strazicich UnitRoot test, Gregory-Hansen cointegration test, Fisher-Seater test, LMN, long run money neutrality, LMSN, long run money superneutrality, LRN, long run neutrality, LRSN, long run superneutrality, PP, Philips Peron, One of the unit root tests, ADF, Augmented Dickey Fuller, One of the unit root tests, KPSS, Kwiatwoski Pilips Schmidt Shin, One of the unit root tests

## Abstract

This paper investigates the long-run money neutrality (LMN) and long-run money superneutrality (LMSN) hypothesis for both the industry sector and the entire Iranian economy by using the data of 1979–2018 and applying Fisher and Seater's (1993) ARIMA framework. Conventional unit root tests, including PP, ADF, and KPSS, are applied to determine the order of integration of variables; however, since the structural break in variables is not considered in these methods, Lee-Strazicich and Zivot-Andrews methods are also applied to take it into account. The findings of money neutrality investigation in the Iranian industry sector show that when the monetary base is the criterion, money neutrality is confirmed, but when liquidity and money volume are the criteria, money neutrality is rejected. Also, the neutrality of money is accepted considering all three monetary aggregates (M1, M2, and M3) in investigating the entire economy. It is not feasible to examine the superneutrality of money since unit root tests confirm that all the variables are I (1).

• As there is more than one structural break in the time series of the study, applying the Lee-Strazicich unit root test has made the results more reliable.
• Neutrality of money testing is not efficient in the case of cointegration between model variables. Thus, the Gregory-Hansen test, which investigates cointegration considering the structural break, is applied.
• The results of this research can guide policy-makers.
• Non-neutrality of money in the industrial sector shows the positive effect of monetary policy on this sector when considering the probability of destructive effects on other sectors.

## Graphical abstract

**Figure:**
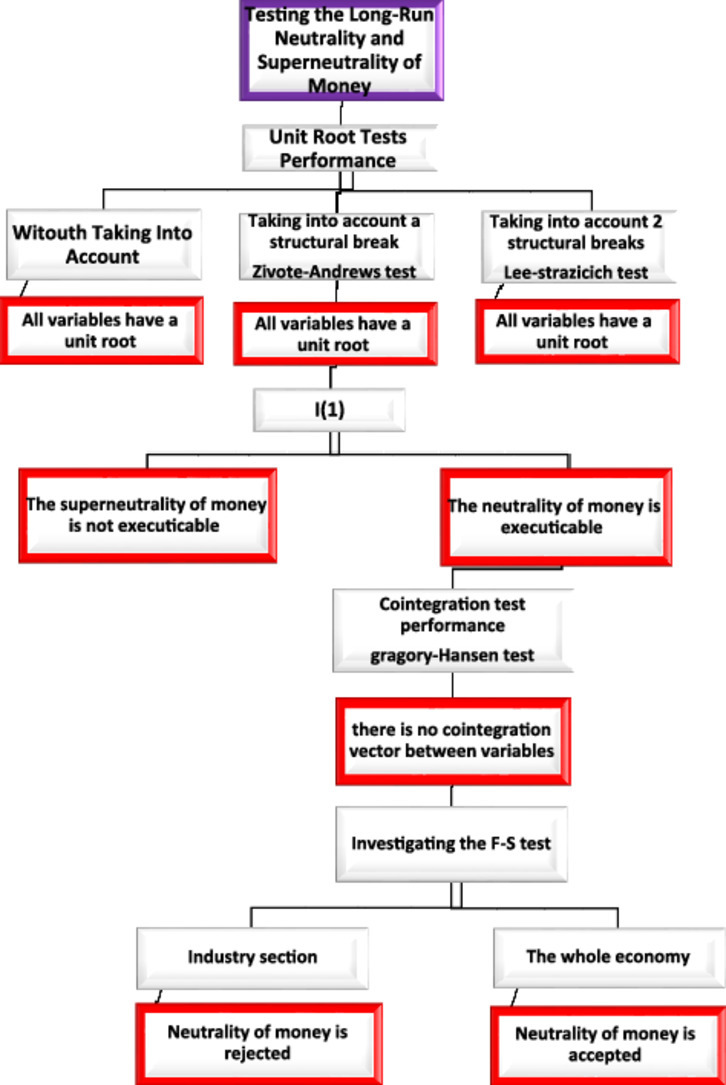

Specifications table

**Table:** 

Subject Area	Macroeconomics
More specific subject area	Neutrality of money, superneutrality of money
Method name	Fisher and Seater methodology
Name and reference of original method	Fisher, M. E, Seater, J. J [16] long run neutrality and superneutrality in an Arima framework. The American economic review. 83(3). 402–4115.
Resource availability	Fisher, M. E, Seater, J. J [16] long run neutrality and superneutrality in an Arima framework. The American economic review. 83(3). 402–4115. Wallace, H.F, Shelley, L. G [53]. Long Run Neutrality of Money in Mexico. economía Mexicana NUEVA ÉPOCA. XVI (2). 219–238 Direct submission
Direct submission or co-submission	

## Introduction

Choosing appropriate policies and tools to eliminate inequality, create stability, and increase economic growth and development is an important issue in macroeconomics. Monetary policies are a kind of policy employed by the central bank to achieve goals, e.g., controlling inflation, creating a proper condition to increase production and employment to the potential level, and preserving the value of the national currency. These policies can accomplish many economic goals, e.g., stimulating economic growth and promoting employment and price stability by affecting the money supply and interest rate [3]. However, the effectiveness or ineffectiveness of monetary policies (known as the neutrality of money in the economy) in real variables is one of the most controversial economic issues.

Neutrality of money originates from the quantity theory of money which states that an exogenous change in the money volume by money authorities in the long run can change the price levels and other nominal variables in the same proportion without any changes in real variables. There are two different but close branches of research on the neutrality of money, which are distinguished by using Barro's definition. Barro [6] states that there will be neutrality of money if a change in the money volume cannot affect the real economic variables such as GDP, although it affects the nominal variables. Barro's definition of the superneutrality of money also states that a change in the monetary growth pattern has no effect on the real variables over time. Neutrality of money has been studied by economists for a long time. Neutrality of money, based on the direct relationship between general price levels and the amount of money in circulation, is so important that is has been discussed since the 16th century and after the publication of Jean Bodin's manual.

According to the neoclassical perspective, only employing unexpected monetary policies can affect the real part of the economy. Monetarists who believe in adaptive expectation formation declare that monetary policies can be effective as long as the expectations are not completed. Thus, both of these schools believe in the effectiveness of monetary policy only in the short run; nevertheless, these properties are mostly related to developed countries and less to developing countries which are affected by monetary policy through bank loans. Moreover, developed countries are more sensitive to transmission mechanisms (interest rate, credit supply, and more) in comparison to developing countries. The availability of too much information, the symmetric nature of information, and the development of financial markets are all factors which bring about an accurate prediction of economic factors, accelerate expectation adaptation, and thus neutralize the monetary policies in the long run [21].

Tobin [52] does not support the superneutrality of money theory which is based on the neoclassical models of Solow [47] and Swan [49] in which it is assumed that, firstly, the money supply increases at a constant rate over time, and secondly, in the steady state, the real money balance is constant over time; given these two conditions, the results of superneutrality of money are obtained.  According to Tobin, as the inflation rises, the households' demand for money alternative assets increases, and therefore money demands decrease. As a result, Tobin claims that inflation increase causes the accumulation of capital and economic growth [13]. In addition to Tobin, studies by Brock [8], Carmichael et al. [9], and Smith et al. [46] do not support the superneutrality of money. On the other hand, Dornbush and Frenkel [14], criticizing Tobin's theory, showed that the Tobin effect simply disappears when the inflation has a positive effect on consumption and, as a result, money is neutral.

Sidrauski [45] does not reject the neutrality of money and claims that money supplies do not have a long-run effect on capitalization and production increase [13]. Studies by Lucas and Haug [31], Coe and Nason [10], Bernanke and Mihov [7], and Weber [54] support the neutrality of money.

Moreover, Fisher and Seater [16] demonstrate that, sometimes, there is some interpretative evidence that is totally different from neutrality and superneutrality of money theories. Thus, its acceptance or rejection completely depends on the statistical population under study. Therefore, the results of neutrality and superneutrality of money studies differ across countries regarding the economy, transparency, and development of those countries. Therefore, it is necessary to investigate the neutrality of money in the economy of every country because it is costly to employ monetary policies, and there are other costs such as inflation in the future. As a result, it is very important to examine and provide exact information on the effectiveness or ineffectiveness of the monetary policy before adopting it.

The industry is one of the important sectors of the economy in every country, and many experts believe that industrial development is essential for sustainable economic growth and development. Therefore, due to the significant effects of the industry sector on macroeconomic variables such as production, consumption, investment, employment, and export, it is essential to pay special attention to this sector. Moreover, since there is two-sided and effective communication between subsectors in the industry and other sectors of the economy, one should pay attention to the impact of different policies on the real production of this sector.  Accordingly, the aim of this study is to answer the following questions:

Is money neutral in the long run in the entire economy? Is money superneutral in the long run in the entire economy? Is money neutral in the long run in the industrial sector? Is money superneutral in the long run in the industrial sector?

Based on the above questions, this paper is organized as follows: Section 2 presents the theoretical foundations of the study; first, the neutrality of money is investigated based on the perspective of different schools, and then the monetary variables and the industry sector of the Iranian economy are investigated. At the end of this section, the studies on neutrality and superneutrality of money in Iran and other countries are reviewed. In Section 3, Fisher and Seater's econometric framework is delineated. In Section 4, after describing the data, the time series properties and empirical results of the study are reported. Finally, Sections 5 and 6 are devoted to discussion and conclusion, respectively.

## Theoretical foundations and literature review

### Money in different schools

Classical school:

According to the classical school theory, the economy is divided into two sectors, a real sector and a monetary sector. Based on the quantity theory of money, MV=PY, where M is the quantity of money, V is the velocity of money, P is the general price level, and Y is the total output of production. Classics state that, assuming the velocity of money and production to be stable, if the volume of money increases by X% in the economy, the general price level will increase by the same amount. Thus, money is neutral in the classical theory and only affects the nominal sector of the economy [50]. Actually, classics believe that all of the economic operators, whether consumer or producer, are never affected by money illusion and organize their economic activities according to real variables; consequently, money volume changes only affect prices, and money is a neutral variable in determining the real performance of the economy. Therefore, the real variables of the economy, e.g., real interest rate, real exchange rate, production, employment, and unemployment are determined regardless of money and money changes. Moreover, the volume of money and its changes determine the level of price and inflation and are effective in the determination of other nominal variables [20].

Keynesian school:

Keynes believed that the quantity of money theory has been extreme in explaining the way that monetary policies affect the total demand. According to him, the classical economists ignored a reality based on which money belongs to the group of assets. This problem resulted in the formation of a new theory of money demand. Keynes introduced three types of motivation related to the money demand and the tendency to maintain the money: trading, speculation, and precautionary. The latter plays an important role in the transmission of monetary policies to the real sector. Overall, it can be stated that Keynesians (except for extreme Keynesians) believe in the effect of monetary policies and their non-neutrality. Furthermore, their analysis does not differentiate between long-run and short-run effectiveness [17].

Monetarism school:

Monetarists hold that nominal shocks may affect the real variables of the economy in the short run, but they definitely do not affect them in the long run. According to this point of view, the classical dichotomy between real and monetary sectors is true in the long run, but this theory is not valid in the short run. Thus, the monetarism school supposes money neutrality in the long run. Monetarists generally believe in three characteristics of money:

1. They emphasize the self-balancing feature of the economy.
2. They hold that the volume of money is a key factor in nominal GDP determination.
3. They have an interventional notion of macroeconomic policy, and their ideas are rooted in classic economy (the schools of Ricardo and Adam Smith) [29].

New classical school

According to the analysis of new classical economists, economic policy-makers cannot affect production and employment by an anticipated money supply increase. In fact, Lucas (1972) [56] showed that in the presence of rational expectations, an unanticipated money supply in the short run can affect the level of economic activities. His studies demonstrated that money is superneutral in the long run, but unanticipated monetary shocks are effective in the short run by creating a liquidity effect on the economy.

Austrian school

According to Austrian school economists, not only does the money supply change, but also the way that money enters the system and finds its way to the economic system affects real variables and economic performance. Supporters of the Austrian approach are opposed to government intervention in the economy and believe that these interventions will exacerbate the situation. This school assumes that money is endogenous to production. The money volume increase is a response to the increased production and due to individuals' and investors' need for more money. In this school, employing monetary policy is not a solution for increasing the production level [2].

New Keynesian school

New Keynesians believe that due to the imperfect competition in the labor market, product and credit (real and nominal adhesion and common credit constraints in the economy), and the convexity of the aggregate supply curve, there is a rational expectation hypothesis and money fluctuations can affect real production, but the impact of the shocks is asymmetric [11].

Real business- cycle school

This school was expanded by Kydland and Prescott as classical economists. It states that nominal variables such as money supply and general price levels cannot affect real variables and real factors' fluctuations; rather, this can only be explained by real changes in the economy [50]. In fact, the observed correlation between money and production does not indicate the effect of money changes on production, but shows the effect of production changes on money, contrary to the common belief [35].

**Figure:**
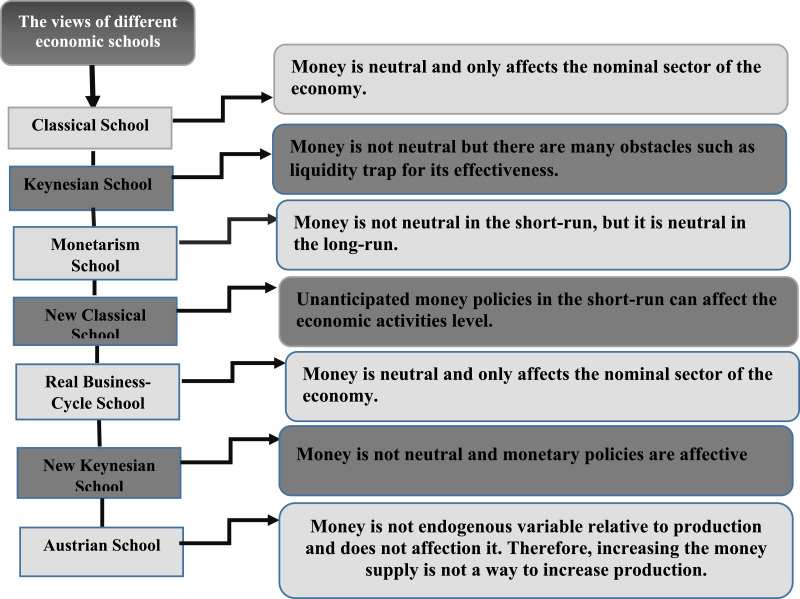

### Monetary variables in Iran

In accounting, liquidity is defined as the sum of debt-side items or the sum of asset-side items of the banking system. Thus, liquidity can be considered either as the investment (debt side) or accreditation (asset side) of the banking system. The debt side includes two components, money and quasi-money, which are basically the same in the process of making money. Nevertheless, the asset side includes two components which are different in nature and indicate money supply consumption, and especially, the credits granted [36].

Money volume and liquidity changes are investigated from two aspects. The first viewpoint is that central bank authorities change the money volume exogenously and intentionally in order to affect and stimulate real production, which is called active monetary policy. The second viewpoint is endogenous money volume changes which are made in response to the real sector shocks or to cover budget deficits by the central bank. Thus, these money volume and liquidity changes are not active monetary policies, but are the logical reaction of the central bank to the shocks specified to very open economies.

One of the most serious problems in developing countries is the control of liquidity and inflation. If liquidity increase is more than the production level, it will cause high inflation. On the one hand, inflation removes financial resources from productive activities and causes brokerage activities; on the other hand, it increases the inequality of income and wealth in society, while it is possible to create financial stability and, as a result, to develop productive activities by liquidity and inflation control in the country.

The Iranian economy has been facing a decline in the central bank's independence, especially in the post-revolutionary years, which has made the central bank obey governmental orders. Thus, when the government has a deficit, it increases the monetary base and, thus, increases the liquidity trend. Consequently, the central bank's independence is very important in controlling the liquidity and money supply.

Lack of access to adequate finances and liquidity has been a major limitation to the production sector from the perspective of enterprise in recent years. Most economic analysts suggest that high liquidity and bank credit overgrowth are the reasons for economic problems, especially double-digit inflation. Excess liquidity at the macro level and lack of liquidity at the micro-level have been known as the "liquidity puzzle". Thus, the liquidity puzzle is made of some micro- and macro-structural factors which have caused liquidity to be inefficient despite its high volume and not circulate among the economic sectors; they have also caused economic shocks which not only increase the enterprise need for working capital, but also disable the former mechanisms for short-term financing [35].

### Industry sector in Iran

The industry sector is one of the basic sectors of the economy which plays a key role in the boom and bust cycles' determination. The industry sector in Iran has several strengths, the most important of which are the abundance of educated job-seekers, proper infrastructure for development, huge energy reserves in the country and low-cost access to them, abundant mineral and industrial resources, production capacity, and comparative advantages in producing a variety of products to develop non-oil exports.

According to the report of the Statistical Center of Iran, the share of the mining and industry sector in Iran's gross domestic product (GDP) is almost 26% in different years, of which the industry sector has the largest share (60%).

Petrochemicals, steel, automobile, and cement are the largest industries in Iran in which government intervention is maximum. These sectors are the most affected by the government's decisions. The government has constantly supported these industries in recent years. Based on diagrams (1) and (2), the production of these industries has been increasing during the period of the present research.

According to the report of the Central Bank of Iran, the employment share in the industry sector is 35.1% on average, which has the second place among the service and agricultural sectors with shares of 48.6 and 16.3%, respectively. In addition, according to the latest report, almost 21% of the investment has been made in the industry sector, and 22.5% of energy consumption in Iran belongs to this sector [3].

**Fig. 1:**
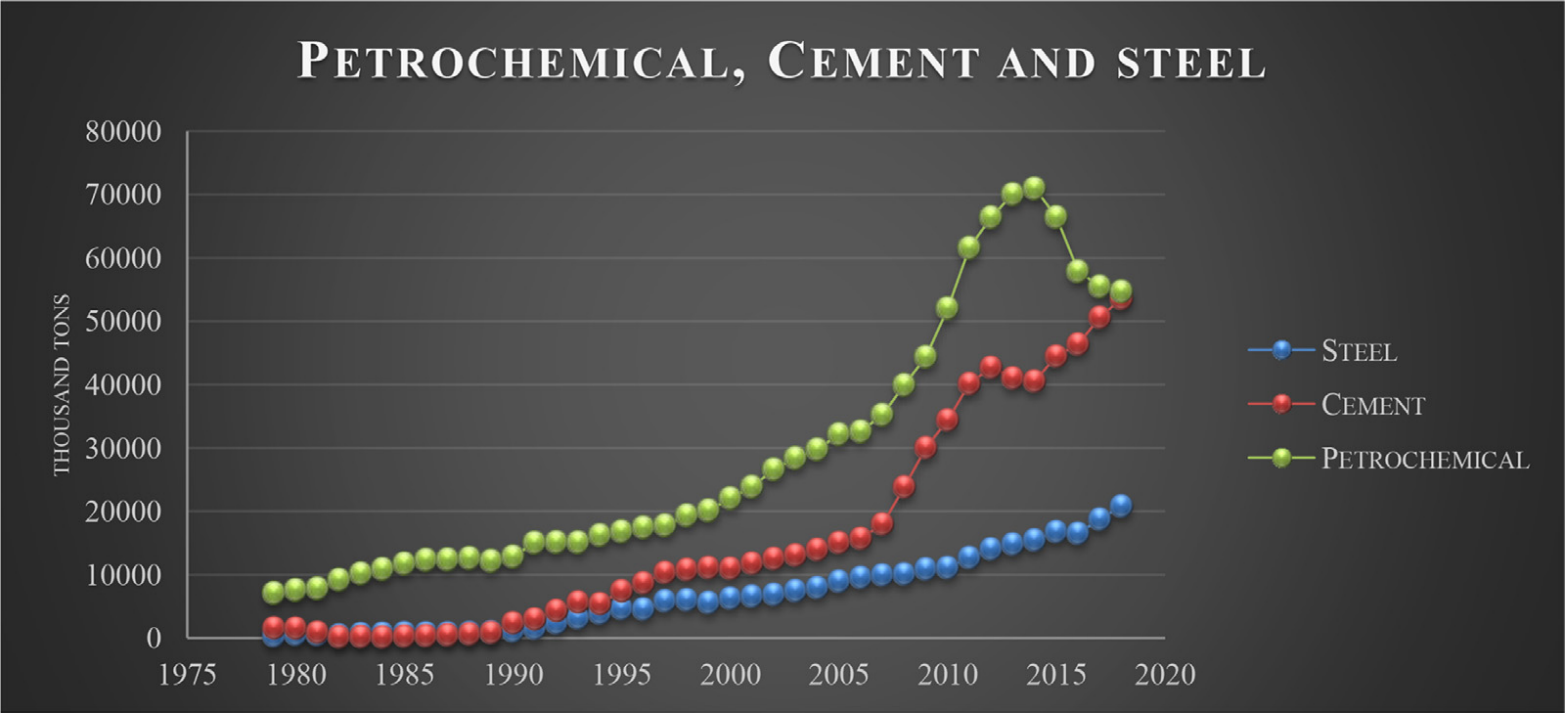
Selected industrial and mining production.

**Fig. 2:**
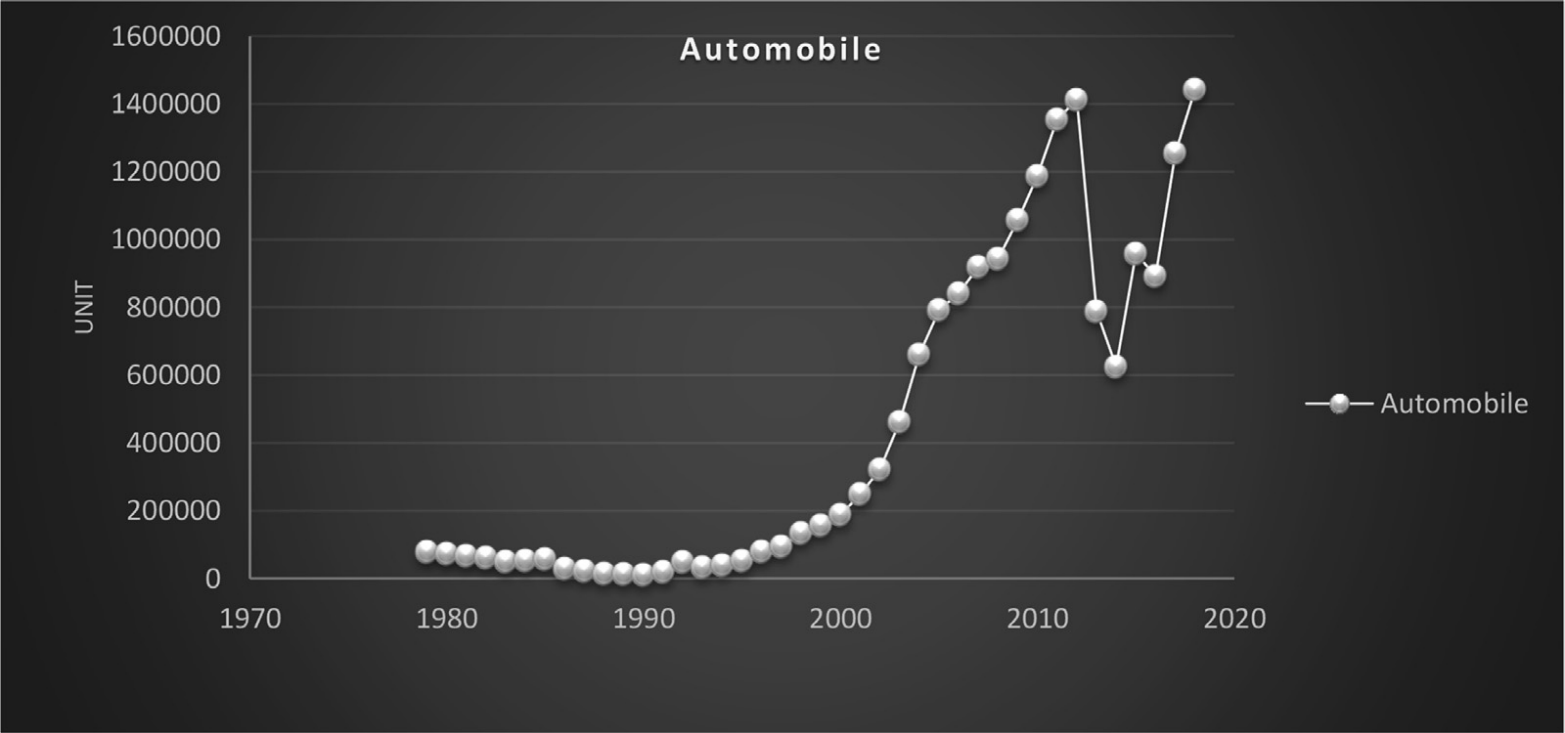
Selected industrial and mining production.

The industry sector in the Iranian economy connects the other sectors, and its growth can be transferred to other sectors. Nowadays, when countries, especially developing economies, pay attention to industrialization by using new technologies, the industry sector can pave the way to achieving this goal by using technical knowledge and implementing knowledge-based economies. Moreover, the experience of developed countries shows the direct link between living standards (increase in per capita income) and industry growth in these countries [24],[39] (Fig. 1 and 2).

### Literature review

In terms of the neutrality of money, Sidrauski [45] examined whether growth rates affect real variables. Evidence indicates that the level of capital stock and actual consumption is independent of the inflation rate and money growth rate. Jefferson [23]^1^investigated the neutrality of money by dividing money into outer and inner parts for the years of 1900–1992 using the VAR model. The result shows that money is neutral, although its impact is limited. Sanchez Fung [43] examined the long-run money neutrality (LMN) and long-run money superneutrality (LMSN) for the Dominican Republic. After analyzing the integration and cointegration properties of the data and implementing the Fisher and Seater [16] method to test LMN and LMSN, it was concluded that money is neutral with respect to prices and superneutral with respect to real GDP.

Samimi and Erfani [42] applied the Fisher-Seater methodology to investigate the long-run neutrality and long-run superneutrality of money for the Iranian economy during the period of 1959- 2002. They found that money is neutral, but superneutrality was not approved. Telatar and Cavusoglu [51] examined the LMN and LMSN using the data of high-inflation countries (Argentina, Brazil, Ecuador, Mexico, Uruguay, and Turkey); it was found that money is neutral in all these countries, except for Ecuador, and superneutrality of money was rejected for Argentina and Uruguay. Wallace and Shelley [53] examined the LMN using Fisher-Seater methodology for 10 selected industries in Mexico. The evidence indicated that LMN can be rejected for up to five of the 10 studied industrial sectors. These findings demonstrated that the effects of monetary policy may differ across sectors, even in the long run.

Puah et al. [38] investigated long-run neutrality (LRN) and long-run superneutrality (LRSN) via the Fisher-Seater method using the data of 10 Southeast Asian countries. The result indicated that the LRN can be excluded from developing economies; monetary extension can have a long-run impact on real output in the economy of Taiwan, Indonesia, and Thailand, but the LRSN is neither confirmed nor rejected. Sulku [48] examined the long-run neutrality of money in the Turkish economy using the Fisher-Seater test, during the period of 1987:Q1–2006:Q3. It was found that money is neutral in Turkey when considering different monetary aggregates, M1, M2, M2Y, and M3.

Hoang [19] explored the relationship between key macroeconomic variables such as inflation, GDP, budget deficit, money supply, and interest rates, by using a structural vector auto-regression (SVAR) model. The results indicated that monetary growth has had a positive impact on inflation in Vietnam, and that inflation has been increasing for three months in response to a positive shock to monetary growth and had no impact on production, budget deficit growth, and interest rate. Sam and Geetha [41] examined the long-run behavior of monetary aggregates towards the Malaysian economy based on quarterly data ranging from 1996 to 2014. In this paper, the unanticipated money supply was tested under the vector error correction model (VECM). The results showed that there is little evidence to support the view of the neutrality of money in Malaysia.

Deev and Hodula [12] examined the superneutrality of money in 29 European economies, and focused on endogenous growth theories such as the Mondel-Tobin model. The model estimation was performed via SVAR, and the methodology was the Fisher-Seater approach. The effects of constant inflationary shock on production growth was investigated by using these models. The results showed that money is superneutral for most countries. Najafi et al. [35] applied the Fisher-Seater methodology to examine long-run neutrality and long-run superneutrality of money in the service sector of the Iranian economy during the period of 1973- 2013. Based on the findings, LRN was rejected in the service sector, and LRSN could not be examined. Finally, Izadkhasti [22] analyzed the impact of monetary policy on inflation and welfare in the dynamic general equilibrium model for the Iranian economy and concluded that, by reducing the growth rate of money supply, labor force production, per capita consumption and production are not changed uniformly, and thus money is superneutral.

## Econometric framework

The Fisher–Seater model

Fisher and Seater introduced a long-run derivative to formulate LRN (long-run neutrality) and LRSN (long-run superneutrality). for this purpose, they assumed a system of log-linear, stationary, and invertible ARIMA model of two variables, such that (m) is the log of money supply and (y) is the log of real GDP. Thus, Eqs. (1) and (2) are the starting points of this system.

(1) $$a\left(l\right)\Delta^{\langle m\rangle }m_{t}=b\left(l\right)\Delta^{\langle y\rangle }y_{t}+u_{t}$$

(2) $$d\left(l\right)\Delta^{\langle y\rangle }y_{t}=c\left(l\right)\Delta^{\langle m\rangle }m_{t}+w_{t}$$

where <m> and <y> are the orders of integration of the money supply and the real GDP, respectively; and u_t_ and w_t_ are independently and identically distributed disturbance terms with zero mean and Ʃ covariance matrix, of which the elements are σ_uu_, σ_uw_ and σ_ww_. The long-run response of the output to a permanent change in money is given by the long-run derivative (LRD_y, m_).

(3) $$LRD_{y,m}=\lim_{K\to \infty }\left[\frac{\partial \left(y_{t+k}/\partial u_{t}\right)}{\partial \left(m_{t+k}/\partial u_{t}\right)}\right]$$

When$\lim_{K\to \infty }\frac{\partial m_{t+k}}{\partial u_{t}}=o$, there are no permanent changes in the monetary variable, so LRN cannot be tested, and this is a special mode. Therefore, the long-run effect can be tested when$\lim_{K\to \infty }\frac{\partial m_{t+k}}{\partial u_{t}}\neq o$, and this indicates that money should be non-stationary in level〈m〉≠0.

Fisher and Seater demonstrated that, if 〈m〉≥1, then Eq. (4) can be written as:

(4) $$LRD_{y,m}=\frac{{\left(1-L\right)}^{\langle m\rangle -\langle y\rangle }\gamma \left(L\right)|L=1}{\alpha \left(1\right)}$$

Where γ(L)andα(L) are the functions of the ARIMA model with two variables, Eq. (1) and Eq. (2).

(5) $$\alpha \left(L\right)=\frac{d\left(L\right)}{\left[a\left(L\right)c\left(L\right)-b\left(L\right)c\left(L\right)\right]}$$

(6) γ(L)=(c(L))/[a(L)c(L)−b(L)c(L)]

Eq. (4) shows that the value of LRD_y,m_ depends on <*m*>- <*y*>:

• If $\langle m\rangle -\;\langle y\rangle \geq 1,\;LRD_{y,m}=0$
• If $\langle m\rangle -\langle y\rangle =o,\;LRD_{y,m\;}=\;\frac{\gamma (1)}{\alpha \;(1)}=\frac{c(1)}{d(1)}$
• If $\langle m\rangle -\langle y\rangle =-1\;,\;LRD_{y,m}$ is defined, only when γ(1)=0, that in this case:
  $$LRD_{y,m\;}=\;\frac{{\left(1-L\right)}^{-1}\gamma \left(L\right)}{\alpha \left(L\right)}=\;\frac{C^{*}\left(1\right)}{d\left(1\right)}\;that:\;c^{*}\left(1\right)=\;{\left(1-L\right)}^{-1}c\left(L\right)$$

They proposed four cases for every hypothesis of long-run neutrality and superneutrality of money:

First, if LRD_y,_*_m_* = 1 and when variable y is nominal, or if LRD_y,_*_m_* = 0 and when variable y is real, then the long-run neutrality of money is resulted. In this case, four equations are separable:

1. 1.〈m〉<1; in this case, LRD is not defined, which shows that permanent changes in money volume have not occurred. Thus, monetary data do not give any information about the long-run neutrality of money.
2. 2.〈m〉≥〈y〉+1≥1, LRD= 0; in this case, because variable y does not show permanent changes while money volume level has been facing permanent shocks, if y is the nominal variable, the theory of neutrality of money in the long run will be rejected; otherwise, it cannot be rejected.
3. 〈m〉=〈y〉≥1;in this case, it is possible to test the hypothesis of LMN. This case has been used in most experimental studies, and the ordinary least squares (OLS) method is usually applied to estimate the coefficient of the model to determine if there is a correlation between permanent changes in money volume and permanent changes in real production.
4. 〈m〉=〈y〉−1≥1. This case is a little more sophisticated as money is neutral in the long run, provided that permanent changes in money do not change the real production growth rate.

Second, if$\;LRD_{y,\Delta m}=1$, and when variable y is nominal, or if$\;LRD_{y,\Delta m}=0,$ and when variable y is real, money is superneutral in the long run. Four models are separable in this case:

1. 〈Δm〉<1; in this case, LRD is not defined because there is no evidence of permanent changes in the money growth rate. Thus, monetary data do not give any information about the superneutrality of money in the long run.
2. 〈Δm〉≥〈y〉+1≥1; in this case, LRD is equal to zero. It means that although there are permanent changes in the growth rate of money volume, no changes in the real production level have been observed, and so the hypothesis of superneutrality of money is confirmed.
3. 〈Δm〉=〈y〉≥1;in this mode, it is possible to test the hypothesis of the superneutrality of money.
4. 〈Δm〉=〈y〉−1≥1;in this case, it is possible to test $LRD_{y,\Delta m}=0$, which means that it can be determined whether permanent changes in the growth rate of money volume are accompanied by permanent changes in the growth rate of real production or not.

The restrictions of the different modes of LRN and LRSN are summarized in Table 1.

**Table 1:** long run neutrality and super neutrality restrictions.

	LRD_Y,M_			$\mathrm{LR}D_{Y,\Delta M}$		
	$\mathrm{LRN}\to \mathrm{LR}D_{Y,M=\omega }$			$\mathrm{LRSN}\to \mathrm{LR}D_{Y,\Delta M}$		
〈Y〉	〈m〉=0	〈m〉=1	〈m〉=2	〈m〉 = 0	〈m〉=1	〈m〉=2
0	undefined	=0	=0	undefined	undefined	=0
1	undefined	c(1)/d(1)	=0	undefined	undefined	c(1)/d(1)
2	undefined	$c^{*}\left(1\right)/d\left(1\right)$	c(1)/d(1)	undefined	undefined	$c^{*}\left(1\right)/d\left(1\right)$

Assuming that money is exogenous in the long run, Fisher and Seater state that b_k_ can be used as an estimator of *c(1)/d(1)* regression (OLS) in Eqs. (7) and (8).

(7) $$y_{t}-y_{t-k-1}=a_{k}+b_{k}\;\left(m_{t}-m_{t-k-1}\right)+e_{kt}$$

(8) $$y_{t}-y_{t-k-1}=a_{k}+b_{k}\;\left(\Delta m_{t}-\Delta m_{t-k-1}\right)+e_{kt}$$

The significant amounts of b_k_ in the above equations indicate the rejection of the neutral theorem and the rejection of the superneutrality of money theorem in the long run.

## The data and test results

### The data

**Fig. 3:**
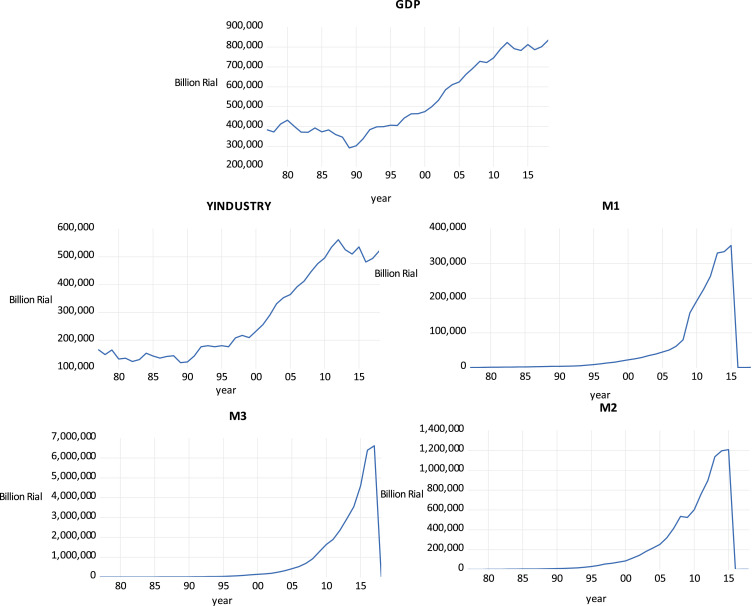
Chart for model variables.

The annual time series data from 1979 to 2018 are used the variables are extracted from the Central Bank of the Islamic Republic of Iran and from the Management and Planning Organization of Iran and include:

GDP at base price 2004
Real production of the industry sector (Y_I_) at base price 2004
Monetary base (M_1_): The monetary base in a country is the total amount of banknotes and coins. This includes the total currency in circulation, plus the currency that is physically held in the vaults of commercial banks, plus the commercial banks' reserves held in the central bank.
Volume of money (M2): is equal to notes and coins in circulation, plus sight deposits.
Liquidity (M3): is equal to the volume of money plus quasi-money (non-sight deposits with banks) (Fig. 3).

### Unit root tests

To apply the Fisher-Seater approach, it is necessary to first investigate the cointegration degree of variables. Thus, different tests in the unit root literature are used. In this sector, whether the variables are stationary is tested by using different tests regardless of the break, considering one break, and considering two structural breaks. The statistical significance level is set at 5% in all the tests.

#### Unit root tests without structural breaks

Common unit root tests are used in this sector in order to investigate whether the variables are stationary, such as Philips-Perron (PP), augmented Dickey-Fuller (ADF), and Kwiatkowski-Phillips-Schmidt-Shin (KPSS). According to Table (2) the existence of the unit root hypothesis is not rejected in any of the variables, and the cointegration degree of all of the variables is equal to 1 (Table 2).

**Table 2:** Unit Root tests without structural break.

Levels				First differences			
Variables	ADF	PP	KPSS	ADF	PP	KPSS	CONCLUSION
GDP	0.09 (−2.93)	−0.04 (−2.93)	0.74 (0.46)	−5.13 (−2.93)	−5.30 (−2.93)	0.15 (0.46)	I(1)
Y_I_	0.77 (−2.93)	0.75 (−2.93)	0.74 (0.46)	−6.55 (−2.91)	−6.64 (−2.93)	0.19 (0.46)	I(1)
M1	−1.87 (−2.93)	−1.87 (−2.93)	0.48 (0.46)	−6.33 (−2.93)	−6.22 (−2.93)	0.10 (0.46)	I(1)
M2	−1.80 (−2.93)	−1.80 (−2.93)	0.50 (0.46)	−6.26 (−2.93)	−6.26 (−2.93)	0.09 (0.46)	I(1)
M3	−1.82 (−2.93)	−1.82 (−2.93)	0.52 (0.46)	−6.43 (−2.93)	−6.43 (−2.93)	0.10 (0.46)	I(1)

The figures in parentheses are t statistic and denote statistical significance at 5 percent levels.

#### Unit root tests with structural breaks

The classical unit root tests such as ADF and KPSS give misleading results in case of structural break existence in the economic variables. Structural changes are highly important in analyzing macroeconomics time series. There can be various reasons which lead to structural changes in time series, such as economic crisis, framework change, political changes, and even regime change. If a structural break is not considered in the time series trend, it may make the estimate results biased towards the non-rejection of the unit root test [37].

As we face different incidents during the period of the study, such as revolution, war, sanction, and inflation, the presence of a structural break is very likely. Thus, in order to avoid misleading results, the unit root test considering structural breaks is applied to investigate whether the model variables are stationary.

Therefore, the Lee-Strazicich is applied, in addition to the Zivot-Andrews test as the unit root test with structural break. The advantage of applying Lee- Strazicich test in comparison to the Zivote-Andrews test, which has been used in previous studies, is that the Lee-Strazicich test considers more than one structural break which is more compatible whit the economy of Iran due to plenty of changes and crises in the Iranian economy in recent years, which result in data structure break.

##### Zivot-Andrews unit root test

Zivot-Andrews is a unit root test with a structural break. The important feature of the method is that there is no need to determine the structural breakpoint. This test identifies the point of structural break and then runs the unit root test accordingly. Non-rejection of the null hypothesis implies that the series is non-stationary, whereas the rejection of the null hypothesis shows that the time series is stationary (Table 3).

The results of the Zivot-Andrews test in Table (3) show that all dependent and independent variables are non-stationary at level, but the first difference is stationary. Thus, all of the model variables are stationary at the first difference considering a structural break. The results of the Zivot-Andrews test according to two models (trend) and (trend and intercept) are the same.

**Table 3:** Zivot-Andrews unit root test.

Levels				First differences			
Variables	T statistic	Break point	lag	T statistic	Break point	Lag	CONCLUSION
GDP	−2.84	2002	0	−5.80	1991	0	I(1)
Y_I_	−3.26	2002	3	−6.40	2011	0	I(1)
M_1_	−3.78	2013	4	−10.13	2009	2	I(1)
M_2_	−1.48	2010	3	−7.41	2010	2	I(1)
M_3_	−1.51	2012	4	−8.10	2011	4	I(1)

The critical values of the variables are at the level of 1%.5% and 10%, −5.34, −4.93 and −4.58.

##### Lee-Strazicich unit root test

A structural break is endogenous in the Lee-Strazicich unit root test (2003), and the existence of two breaks in both the null and alternate hypothesis is possible. The data generation process in this test is as follows:

(9) $$y_{t}=\delta^{\prime }\;z_{t}+e_{t\;}$$

Where z_t_ is an exogenous variable vector and$\;e_{t}=\beta_{1}e_{t-1}+\varepsilon_{t},\;\varepsilon_{t}∼NIID\left(0,\delta^{2}\right)$. In this model, by considering two breaks in the intercept, z_t_ is defined as follows:

(10) $$z_{t}={\left[1,t,D_{1t},D_{2t}\right]}^{\prime }$$

Where $t\geq T_{\beta j}+1$ if *j* = 1, 2 and$\;D_{jt}=1$; otherwise its value will be zero. $T_{\beta j}$ indicates the time of break occurrence. The two-break LM unit root test statistics are obtained from the regression estimate based on LM as follows:

(11) $$\Delta y_{t}=\delta^{\prime }\Delta z_{t}+\varphi \tilde{s_{t-1}}+u_{t}$$

Where $\tilde{s_{t}}=y_{t}-{\tilde{\psi }}_{t}-z_{t}\tilde{\delta }$ and *t* = 2 …T. $\tilde{\delta }$ is a coefficient obtained from regression $\Delta y_{t}$ on$\Delta z_{t}$. ${\tilde{\psi }}_{t}$is defined as $y_{1}-z_{1}\tilde{\delta }$ where $y_{1}$and $z_{1}$ indicate the first observation of $y_{t}$and $z_{t}$, respectively.

The null hypothesis is defined as ϕ=0 and is tested by applying the respective t-statistics. The structural breakpoint considering all possible breaks is chosen when the t-statistic has a minimum value.

The critical value in Lee and Strazicich's (2003 and 2004) paper has been investigated and calculated, and the results are reported in Table (4).

**Table 4:** Lee-Strazicich unit root test.

Levels				First differences			
Variables	S_t-1_	TB_1_	TB_2_	S_t-1_	TB_1_	TB_2_	CONCLUSION
GDP	−0.24 (−3.18)	1987	1991	−1.05 (−5.70)	1990	2007	I(1)
Y_I_	−0.21 (−2.96)	1991	2006	−1.43 (−6.21)	2009	2012	I(1)
M1	−0.66 (−2.11)	2009	2012	−1.36 (−6.33)	1996	2012	I(1)
M2	−0.15 (−1.72)	1994	2008	−1.78 (−17.7)	1996	2009	I(1)
M3	−1.82 (−2.93)	1996	2009	−2.93 (−6.43)	1996	2012	I(1)

The critical values of the variables are at the level of 1%.5% and 10%, −4.07, −3.56 and −3.29.

Table (4) illustrates that all of the real and monetary variables are integrated of order one, and all of the model variables are endogenously stationary when considering two structural breaks after one difference.

### Cointegration test

It is essential to investigate the existence of a long-run equilibrium relationship among model variables before testing the neutrality of money. This process is necessary for ensuring the significance of money neutrality testing. Serletis and Koustas (1998) [57] reported that testing for neutrality is not efficient in case of cointegration existence. If there is a cointegration relationship between monetary and real variables of the economy, the hypothesis of the neutrality of money will be rejected, and so there is no need to test the neutrality of money.

#### Gregory-Hansen cointegration tests

Kunitomo [28] states that if there are structural breaks in model variables, the common cointegration tests may result in false cointegration. Thus, considering the period of study over which there were potential structural breaks in the Iranian economy, it is necessary to consider the impacts of these structural breaks to avoid false cointegration.

It is assumed that there is a structural direction change date in the cointegration vector between time series variables in this test. The null hypothesis states that there is no cointegration relationship. The most important advantage of this test is endogenously determining the direction change point between two variables.

Gregory and Hansen used three models: (c) level change model, (C/T) level change model with trend, and (C/S) regime change model, to obtain test statistics. In this test, 70% of the observations of time series are selected, and a dummy variable is defined for those years. Every breakpoint is estimated by applying the OLS method, the residual sentences are calculated, the ADF test is run, and the year with the least ADF statistic is chosen as the structural break year.

This has been performed for all three models, (c), (c/t), and (c/s), and the results are listed in Table (5).

**Table 5:** Gregory-Hansen cointegration test according to model (C/S).

Variables						
	GDP			Y_i_		
	ADF	Z_a_	Z_t_	ADF	Z_a_	Z_t_
M1	−3.40(−4.95)	−14.31(−47.4)	−3.19(−4.95)	−4.32(−4.95)	−19.20(−47.4)	−3.70(−4.95)
Date	2007	2007	2007	2006	2006	2006
M2	−3.23(−4.95)	−14.19(−47.4)	−3.06(−4.95)	−3.56(−4.95)	−18.69(−47.4)	−3.60(4.95)
Date	2008	2007	2007	2007	2007	2007
M3	−3.04(−4.95)	−13.19(−47.4)	−2.97(−4.95)	−3.71(−4.95)	−16.71(−47.4)	−3.39(−4.95)
Date	2008	2007	2007	2006	2006	2006

The figures in parentheses are t statistic and denote statistical significance at 5 percent levels.

Table (5) presents the results of the Gregory-Hansen cointegration test considering structural break and according to model (C/S) (regime change or structural direction change).

According to the three statistics z_a_, z_t_, and ADF, the null hypothesis stating that there is no cointegration and long-run relationship between variables is not rejected. As a result, there is no cointegration vector between monetary and real variables.

Considering that there is no cointegration between monetary variables (M1, M2, M3) and GDP and the real production of the industry sector, it is possible to test the neutrality of money by applying the Fisher-Seater method. The result of the Gregory-Hansen cointegration test according to two models of (C) and (C/T) is attached (see the appendix). The results obtained from these two patterns support the null hypothesis of the test.

### The test results of LRN and LRSN

As evident from the unit root tests, all the variables are I (1); therefore, in the industry sector and in the whole economy, only LMN can be tested based on the methodology. As all the variables' aggregates are integrated of order one (<2), the LMSN test cannot be applied to any of the variables.

In order to investigate the LMN, Eq. (4) whose consistent estimator is coefficient (b_k_) in Eq. (7) is used. Therefore, in this paper, Eq. (7) is estimated once for the industry sector and once for the whole economy for *K* = 1, 2, 3….20, and the results are presented in Tables (6) and (7), respectively (Table 6).

**Table 6:** Estimated bk of Eq. (7) for M1, M2, M3 (industry sector).

variables		*K* = 1	*K* = 2	*K* = 3	*K* = 4	*K* = 5	*K* = 6	*K* = 7	*K* = 8	*K* = 9	*K* = 10
M1	b_k_	−0.01	0.003	−0.04	−0.03	−0.04	−0.09	−0.02	−0.03	−0.10	−0.04
	*t-* statistic	−0.31	0.06	−0.42	−0.55	−0.05	−1.32	−0.36	−0.50	−1.11	−2.04
M2	b_k_	0.06	0.02	0.07	0.05	0.04	0.05	0.05	0.04	0.05	0.01
	*t-* statistic	3.16	1.61	3.61	2.71	2.38	3.06	2.92	2.54	2.75	0.56
M3	b_k_	0.01	0.003	0.01	0.008	0.007	0.009	0.009	0.007	0.009	0.006
	*t-* statistic	3.03	1.09	3.16	2.15	2.21	2.86	2.53	2.54	2.56	0.91

variables		*K* = 11	*K* = 12	*K* = 13	*K* = 14	*K* = 15	*K* = 16	*K* = 17	*K* = 18	*K* = 19	*K* = 20
M1	b_k_	0.01	0.006	−0.01	−0.06	−0.06	0.03	0.03	0.04	0.03	−0.06
	*t-* statistic	0.18	0.008	0.21	−0.73	−0.76	0.42	0.54	0.59	0.42	−0.10
M2	b_k_	0.04	0.06	0.05	0.05	0.05	0.03	0.04	0.06	0.03	0.05
	*t-* statistic	2.03	3.01	3.00	2.74	2.61	1.42	2.40	3.03	2.00	2.15
M3	b_k_	0.008	0.01	0.009	0.009	0.01	0.005	0.008	0.01	0.006	0.009
	*t-* statistic	1.98	2.73	2.65	2.64	2.65	1.32	2.44	2.69	1.70	2.44

Tstatistic denotes statistical significance at 5 percent level.

**Table 7:** Estimated bk of Eq. (7) for M1, M2, M3 (whole economy).

variables		*K* = 1	*K* = 2	*K* = 3	*K* = 4	*K* = 5	*K* = 6	*K* = 7	*K* = 8	*K* = 9	*K* = 10
M1	b_k_	0.05	0.003	0.04	0.03	0.03	−0.002	0.04	0.02	0.04	0.01
	*t-* statistic	2.18	0.14	2.24	1.33	1.18	−0.09	1.54	1.16	1.49	0.90
M2	b_k_	0.03	0.05	0.06	0.03	0.003	0.02	0.03	0.05	−0.07	−0.01
	*t-* statistic	0.67	0.84	0.96	0.39	0.03	0.35	0.48	0.76	−0.57	−0.21
M3	b_k_	0.008	0.001	0.007	0.005	0.006	0.0008	0.007	0.004	0.007	0.003
	*t-* statistic	1.92	0.25	1.96	1.24	1.40	0.16	1.53	1.19	1.50	0.98

variables		*K* = 11	*K* = 12	*K* = 13	*K* = 14	*K* = 15	*K* = 16	*K* = 17	*K* = 18	*K* = 19	*K* = 20
	b_k_	0.02	0.05	0.03	0.03	0.02	0.01	0.03	0.04	0.01	0.03
M1	*t-* statistic	0.94	0.51	1.38	1.52	0.90	0.69	1.38	1.64	0.91	1.07
M2	b_k_	0.024	0.005	−0.04	−0.10	−0.08	0.05	0.05	0.02	−0.09	−0.03
	*t-* statistic	0.24	0.06	−0.45	−0.87	−0.61	0.52	0.61	0.25	−0.89	−0.23
M3	b_k_	0.005	0.008	0.005	0.006	0.005	0.003	0.007	0.007	0.003	0.006
	*t-* statistic	1.02	2.00	1.30	1.70	1.02	0.75	1.49	1.58	0.81	1.27

T statistic denotes statistical significance at 5 percent level.

Based on Table (6), the coefficient (b_k_) for the variable (M_1_) is statistically insignificant, but it is significant for the other two variables (M_2_) and (M_3_). Therefore, in the industrial sector, the neutrality of money is confirmed when the test is performed on the basis of M1. Nevertheless, the neutrality of money theory is not accepted in terms of the other two variables, liquidity and money volume.

Table (7) shows that the coefficient (b_k_) is statistically insignificant for all three variables, (M_1,_ M_2_, M_3_). As a result, the neutrality of money is approved when the whole economy is considered.

Eq. (12), which has been applied by FS, tests the relationship between real production and money without considering the trends in variables. Still, if a trend in variables is confirmed, Eq. (12) should be estimated**.**

(12) $$y_{t}-y_{t-k-1}=a_{k}+\lambda_{t}+b_{k}\;\left(m_{t}-m_{t-k-1}\right)+e_{kt}$$

If coefficient (b_k_) becomes significant, the theory of neutrality of money will be rejected. Regression $y_{t}=\lambda_{0}+\lambda_{1}t+\upsilon_{t}$ is estimated for autocorrelation by the Newey-West method in order to determine the trend of the variables. If the coefficient of the variable is significant, it will be confirmed that there is a trend in the variable. The results in Table (8) demonstrate that all the variables have a positive trend.

**Table 8:** The trend coefficient values and the t-statistic in regression$\;y_{t}=\lambda_{0}+\lambda_{1}t+\upsilon_{t}.$

Variables	T statistic	coefficient $\lambda_{t}$
GDP	3.78	13,175.3
Y_I_	4.83	10,157.5
M_1_	2.19	13,376.2
M_2_	2.11	14,186.5
M_3_	2.36	68,997.5

After confirming the existence of a trend in the variables for the LMN test, Eq. (12) is estimated once for the industry sector and once for the whole economy. The results are given in Table (9) and (10).

**Table 9:** Estimated b_k_ of Eq. (12) for M_1,_ M_2_, M_3_ (industry sector).

Variables		*K* = 1	*K* = 2	*K* = 3	*K* = 4	*K* = 5	*K* = 6	*K* = 7	*K* = 8	*K* = 9	*K* = 10
M1	b_k_	−0.10	−0.03	−0.12	−0.20	−0.09	−0.18	−0.01	−0.09	−0.18	−0.14
	*t-* statistic	01.18	−0.56	−1.77	−1.16	−1.08	−3.03	−0.67	−0.90	−1.83	−4.21
M2	b_k_	0.07	0.02	0.07	0.06	0.04	0.05	0.05	0.04	0.05	0.02
	*t-* statistic	3.39	1.20	3.73	2.77	2.11	3.10	2.66	2.37	2.41	1.67
M3	b_k_	0.01	0.001	0.01	0.009	0.007	0.01	0.009	0.008	0.01	0.001
	*t-* statistic	2.98	1.33	3.42	2.22	2.13	2.79	2.46	2.51	2.52	0.23

Variables		*K* = 11	*K* = 12	*K* = 13	*K* = 14	*K* = 15	*K* = 16	*K* = 17	*K* = 18	*K* = 19	*K* = 20
	b_k_	−0.11	−0.13	−0.13	−0.16	−0.016	−0.08	−0.09	−0.09	−0.13	−0.19
M1	*t-* statistic	−1.24	−1.49	−1.25	−1.28	−2.04	−0.83	−1.05	−0.94	−1.13	−2.06
M2	b_k_	0.05	0.06	0.05	0.05	0.06	0.03	0.05	0.06	0.04	0.06
	*t-* statistic	2.10	3.03	2.93	2.72	2.64	1.52	2.45	2.87	2.01	2.49
M3	b_k_	0.009	0.01	0.009	0.009	0.01	0.006	0.001	0.01	0.007	0.01
	*t-* statistic	2.05	2.79	2.58	2.58	2.63	1.37	2.49	2.55	1.99	2.41

T statistic denotes statistical significance at 5 percent level.

**Table 10:** Estimated b_k_ of Eq. (12) for M_1,_ M_2_, M_3_ (whole economy).

Variables		*K* = 1	*K* = 2	*K* = 3	*K* = 4	*K* = 5	*K* = 6	*K* = 7	*K* = 8	*K* = 9	*K* = 10
M1	b_k_	0.05	0.008	0.05	0.03	0.03	−0.003	−0.04	0.02	0.04	0.01
	*t-* statistic	2.16	0.35	1.49	1.35	1.15	−0.13	−1.63	1.22	1.55	0.97
M2	b_k_	−0.05	0.009	−0.04	−0.04	0.02	0.04	0.02	−0.003	−0.17	−0.14
	*t-* statistic	−0.61	0.11	−0.50	−0.31	0.18	0.38	0.25	−0.03	−1.57	−1.77
M3	b_k_	0.008	0.002	0.008	0.006	0.006	0.001	0.008	0.005	0.008	0.003
	*t-* statistic	1.58	0.54	2.32	1.25	1.36	0.31	1.65	1.27	1.55	1.09

		*K* = 11	*K* = 12	*K* = 13	*K* = 14	*K* = 15	*K* = 16	*K* = 17	*K* = 18	*K* = 19	*K* = 20
	b_k_	0.03	0.05	0.03	0.03	0.03	0.02	0.03	0.05	0.02	0.04
M1	*t-* statistic	1.02	2.11	1.42	1.60	1.07	0.83	1.46	1.69	0.99	1.40
M2	b_k_	−0.06	−0.10	−0.12	−0.15	−0.11	−0.02	−0.02	−0.03	−0.11	−0.05
	*t-* statistic	−0.57	−1.10	−1.51	−2.00	−1.33	−0.23	−0.13	−0.28	−1.31	−0.33
M3	b_k_	0.005	0.008	0.006	0.007	0.006	0.004	0.006	0.009	0.003	0.008
	*t-* statistic	1.08	2.02	1.32	1.76	1.15	0.87	1.58	1.60	0.91	1.55

T statistic denotes statistical significance at 5 percent level.

Based on Tables (9) and (10), when the trend variable is considered in the model, almost the same results are obtained. Therefore, in the industry sector, the LMN is confirmed in the case of monetary base as the criterion, and the LMN is rejected when liquidity and money volume are considered as the criteria.

## Discussion

Liquidity is essential for economic transactions, and lack thereof can decrease the pace of economic growth of countries. On the other hand, a tremendous growth of liquidity and fiat money printing will definitely cause inflation. Liquidity volume control, which is in proportion to production growth, is one of the basic roles of monetary policy. The monetary authorities of a country can control cash flow in society by using this tool and affect economic growth and development by correctly guiding it into productive investment. The literature review shows that the effectiveness or ineffectiveness of liquidity and money has been always the concern of economists and researchers who have tried to investigate the relationship between money volume change, money volume growth rate, and production.

Fisher and Seater [16] stated that if money volume changes in a way that the changes are in proportion to price level changes, even though there is money volume change, real variables such as real production do not change, and this condition will be called money neutrality. Nevertheless, if the money supply changes permanently and the changes keep the real variables' level stable, it will be called the superneutrality of money.

Specialists have frequently investigated neutrality in the whole economy, but whether money volume change can affect real production, specifically in economic sectors, has received less attention from researchers. It is essential to investigate these basics in every sector due to the different nature and structure of economic sectors. Thus, the present study investigated this principle specifically in the industry sector (because of the higher importance of this sector in economic growth increase and productive capacity enhancement), while also investigating the neutrality and superneutrality of money in the whole economy.

.The finding of money neutrality investigation in the whole Iranian economy revealed that money neutrality is confirmed in the long run. Therefore, money volume change by the authorities cannot change real production in the long run. Abbasinezhad and Shafiei [1], Bahar and Rasouli [4], Lashkari and Kashani [29], Khodaparast [25], Komijani et al. [26], and Moradi and Naseri [33] have confirmed the neutrality of money in the whole economy of Iran using different econometric methods. These findings indicate that money changes in the long run affect the price level but does not have any effect on the whole production.

Interpreting the results of their investigation which confirmed money neutrality in developing countries, Telatar and Cavusoglu [51] state that the economic agents in these countries have learned how to live with and hedge against high inflation, making the monetary policy ineffective in the long run. However, the findings of Heydari [18], Khosh Akhlagh et al. [27], and Fallahi [15] have rejected money neutrality in the whole economy of Iran.

The findings of money neutrality investigation in the industry sector of Iran showed that when the monetary base is the criterion, money neutrality is confirmed, but when liquidity and money volume are the criteria, money neutrality is rejected. It means that permanent changes in money volume can affect the real production of the industry sector in the long run. The findings of Motiee and Safdari [34] and Baradaran and Zomorodian [5] are consistent, whereas the findings of Shahbazi and Karimzadeh [44], Monjazeb [32], and Zonouzi, Golipour (2012) [55] are inconsistent with our results.

Najafi et al. [35] state that money non-neutrality in an economic sector is obvious since liquidity increase changes resource allocation; in the other words, new money enters the economy through one sector, and then affects the other sectors. When interpreting money non-neutrality in the industry sector of Mexico, Wallace and Shelley [53] express that the consideration of wealth effects from monetary changes offers the most promising way for explaining the absence of long-run neutrality of money. In fact, the non-neutrality of money in some industries is suggestive of a distributional effect which may be expected from the changes in wealth.

Additionally, the interpretation of this result can be made based on the monetary policy transmission mechanism. According to Laidler [30], the mechanism of monetary policy transmission is a process from monetary policy to nominal income. This process starts with monetary policy and ends with production and prices. There are many channels to transmit monetary policy to different sectors of the economy. However, in Iran, as liquidity is mostly created in the banking system and the industry sector needs more credit and capital to receive from the banks than other sectors, one can claim that credit channels are one of the reasons for the industrial production increase affected by money volume increase.

The bank deposits are increased by employing an expansionary monetary policy, and the banks accept many applications from those who seek credit, so the industries can receive more credit to increase investment and production by more working capital.

Although money is neutral in the whole economy, it can affect the real production of the industry sector; thus, the management of monetary policies by policy-makers is very important. They should provide full supervision for credit expansion in the sub-sectors of the industry. Central bank officials can also bring about an increase in industrial production by adopting expansionary monetary policies depending on the provision of mechanisms to direct liquidity to the production of this sector.

Moreover, it is possible to promote economic growth by reforming the banking system. The profit-seeking behavior of the banks in the Iranian economy has been quite noticeable in recent decades; they often use bank credit creation to pursue their personal interests. The government can direct bank credit creation to development plans and industrial boom by designing a specific incentive system to direct the banks' profit-seeking behavior and centralized allocation of the credits. In general, controlling liquidity and directing it toward sectors that have a capacity for growth but do not have enough credit can ameliorate recession and prevent the inflation caused by liquidity increase. Many economies in East Asia, e.g., Japan, Taiwan, South Korea, and China, achieved economic growth after World War II by implementing a credit-led regime. Iran had a successful experience in this field in the 40 s. In this period, which only lasted six years (1962–1967), the Ministry of Economy promoted and developed the industries by directing the created money toward infant industries using key solutions, which led to an econometric growth of 16% in Iran [40].

In addition to the neutrality of money, the superneutrality of money in the whole economy and in the industry sector of Iran was investigated in this study, an examination which cannot be done based on the results of unit root tests. Studies by Komijani et al. [26] and Moradi and Naseri [33] agree with our findings, while those by Bahar an d Rasouli [4] and Samimi and Erfani [42] reject the superneutrality of money in the whole economy.

In general, there appear to be different results about the neutrality and superneutrality of money in the industry sector and the whole economy of Iran. The methods of money neutrality evaluation and the time period of different studies, in addition to other possible reasons, have probably affected the results.

## Conclusion

This study adopted the FS method to evaluate LMN and LMSN in the whole economy and the industry sector of Iran for the period of 1979- 2018. In this period, LMN and LMSN theory testing depends on the collective degree of monetary variables and real variables of the economy. If the monetary variables are I (0), none of the neutrality of money theories can be tested as there is no permanent change in the monetary variable. If the monetary variables are at least I (1), it is possible to test the above theories. Accordingly, different unit root tests were performed with and without structural break, and their results confirmed that all of the variables are I (1). Thus, based on the Fisher-Seater theory, only LMN could be investigated. The result of the estimated coefficients of the model showed that LMN is rejected in the industry sector when liquidity and money volume are the criteria, and is accepted when the monetary base is the criterion. Still, when the whole economy is investigated, the results proved that LMN is accepted regarding any of these three criteria. Also, according to the result of unit root tests and the confirmation that the variables are I (1), it is impossible to investigate LMSN in the Iranian economy.

The findings of this research can guide policy-makers in adopting monetary policies as neutrality or non-neutrality of money, which is very important in the economy of every country. If, in presence of money neutrality, the authorities use expansionary monetary policies to stimulate production or decrease the unemployment rate, not only will it not have any positive impact on the variables' condition, but it may even result in another disaster for the economy by increasing inflation and price level.

## Suggestions for future research

It is recommended that the neutrality and superneutrality of money of other Iranian economy sectors be evaluated in future studies via different methods. It is also suggested that the anticipated and unanticipated monetary policy effects on every sector of the economy be separately investigated.

## Declaration of Competing Interest

Not applicable

## Figures and Tables

**Table:** 

Variables						
	GDP			Y_i_		
	ADF	Z_a_	Z_t_	ADF	Z_a_	Z_t_
M1	−3.40(−4.95)	−14.31(−47.4)	−3.19(−4.95)	−2.71(−4.61)	−16.48(−40.48)	−3.05(−4.61)
Date	2007	2007	2007	2003	2003	2003
M2	−3.23(−4.95)	−14.19(−47.4)	−3.06(−4.95)	−2.59(−4.61)	−15.59(−40.48)	−2.91(4.61)
Date	2008	2007	2007	2003	2003	2003
M3	−2.84(−4.61)	−16.81(−40.48)	−3.14 (−4.61)	−2.84(−4.61)	−16.81(−40.48)	−3.14 (−4.61)
Date	2008	2007	2007	1999	2003	2003

The figures in parentheses are t statistic and denote statistical significance at 5 percent levels.

**Table:** 

Variables						
	GDP			Y_i_		
	ADF	Z_a_	Z_t_	ADF	Z_a_	Z_t_
M1	−4.73(−5.50)	−21.31(−58.58)	−3.97(−5.50)	−4.90(−5.50)	−23.42(−58.58)	−4.28(−5.50)
Date	2003	2004	2004	2006	2006	2006
M2	−4.67(−5.50)	−22.28(−58.58)	−3.99(−5.50)	−4.47(−5.50)	−22.56(−58.58)	−4.24(−5.50)
Date	1995	1987	1987	2006	2006	2006
M3	−4.40(−5.50)	−19.21(−58.58)	−3.74 (−5.50)	−4.41(−5.50)	−21.17(−58.58)	−4.12 (−5.50)
Date	2000	1987	1987	2006	2005	2005

The figures in parentheses are t statistic and denote statistical significance at 5 percent levels.

## Appendix

Gregory-Hansen cointegration test according to model (C)

Gregory-Hansen cointegration test according to model (c/t)

## Appendix B Supplementary materials

Image, application 1

## Footnotes

1 3 This is part of a thesis on the neutrality test of the Iranian stock market.
